# A Comparison of Participant Demographics Across Co-Designed Recruitment Methods to Two Student Mental Health Trials: Cross-Sectional Observational Study

**DOI:** 10.2196/76018

**Published:** 2026-03-17

**Authors:** Jemima Dooley, Alanis Whitmore, Ella Gillett, Edward Watkins

**Affiliations:** 1 Mood Disorders Centre, School of Psychology, University of Exeter, Henry Wellcome Building for Mood Disorders Research, Perry Rd, Exeter, EX4 4QG, United Kingdom, 44 01392 726449 ext 6647

**Keywords:** social media, trial recruitment, mental health, intervention, diversity

## Abstract

**Background:**

Using social media platforms has been demonstrated to be a successful recruitment method, especially for young people. Two cited benefits of using social media for recruitment are its ability to quickly increase sample size and engage hard-to-reach participants.

**Objective:**

This study aimed to (1) provide a pragmatic depiction of co-designing and implementing a social media strategy with our advisory group and (2) compare demographic information of participants recruited via social media and other methods. Our objective was to provide evidence for future trials to implement social media recruitment with maximum efficiency.

**Methods:**

Participants were 2369 UK university students who consented to take part during the recruitment timeframe of 2 mental health trials. Our student advisory group advised on content, platform, and timing of engagement. We trialed 10 different adverts over a 12-month recruitment period. Descriptive analysis evaluated advert reach and link clicks using Meta/TikTok business data, website traffic using Google Analytics, screening consent, and enrollment using REDCap (Research Electronic Data Capture; Vanderbilt University) software. A cross-sectional observational analysis used chi-square and *t* tests to compare ethnicity, gender, sexual orientation, disability status, and university attended among 842 participants recruited via social media and 1527 participants recruited by other methods.

**Results:**

Through extensive student advisor input, an Instagram carousel advert led to a boost in participant recruitment. However, this fluctuated over the academic year, with numbers dropping completely over the summer months. All tests used *α*=.05. There was a difference in gender among those recruited through social media versus other recruitment methods on campus (*χ*²_2_=8.34, *P*=.02), with social media resulting in a higher proportion of gender-diverse students (27/370, 7% vs 30/711, 4%; 95% CI 3.4%‐13.7%), but fewer male students (35/370, 4%, 95% CI [3.4%‐13.7%] vs 99/711, 7%, 95% CI [1.6%‐9.8%]). Those recruited from social media were younger than those recruited through other methods, with a mean difference of −3.49 years (SE=0.31, 95% CI [–3.94 to –3.04]; *t*_1927.5_=15.146, *P*<.001). A significantly higher proportion of students in the social media sample were from the LGBTQIA+ community (180/351, 51%, 95% CI [41.3%‐60.6%] vs 350/711, 37%, 95% CI [28.2%‐46.8%]; *χ*²_1_=17.87, *P*<.001). There was also a significant difference in the reported disability (103/375, 27.5%, 95% CI [19.7%-37.0%] vs 154/723, 21.3%, 95% CI [14.4%‐30.3%]; *χ*²_1_=4.90, *P*=.03). There was no difference in ethnicity between the 2 groups (*χ*²_1_=2.4609, *P*=.12).

**Conclusions:**

Our study describes how different recruitment approaches influence participant characteristics in clinical trials and highlights challenges in implementing a co-designed recruitment strategy in a university setting. This contributes to the field by providing research evidence on the efficacy of different recruitment strategies for planning future trials. Our key real-world recommendation is to allow time and resources for planning multiple recruitment strategies to ensure a diverse range of participants take part in research.

## Introduction

The modern age presents equally modern issues for researchers in participant recruitment [1]. Social media has proven to be an effective recruitment method, often contributing to higher numbers of study enrollment than traditional, face-to-face methods [2-4]. It enables researchers to quickly reach target populations [5,6], increasing access to hard-to-reach groups, especially regarding socially sensitive research topics [7]. However, there are some concerns that social media recruitment leads to biased samples due to certain demographic groups being more active online [6,8].

There are a variety of metrics that can be used to consider the “success” of a post or advertisement on social media, such as view count (reach), user interaction (likes, shares, and comments), and link clicks. However, when considering using social media for research, there is a differentiation to be made between general outreach as indexed by the view count or interaction, versus targeted outreach to a specific population and the link clicks to a research webpage, which recruitment requires [9]. In terms of general outreach, there is increasing literature outlining how young people access mental health information on social media. Personal, authentic videos have been reported to be most successful [10,11], with evidence of the efficacy of a so-called “influencer” personality in promotion of mental health content [12]. However, increasing research is demonstrating that, if health professionals and academics can implement social media campaigns effectively, following formats demonstrated as successful by other content creators, they can both influence perspectives on health care and increase engagement with evidence-based health care information [13-15].

However, research into what constitutes a successful social media advert specifically for trial recruitment is in its infancy. A recent review of recruitment of young people to digital mental health interventions found that half the studies reviewed did not use social media, while 14% used exclusively social media [16]. However, research shows that online and offline recruitment methods have different levels of success and can impact the diversity of participants, with female participants and those of White ethnicity accounting for higher proportions of samples recruited online [17-19]. This suggests that studies should implement mixed methods in recruitment to lead to diverse samples, but there is a need for more research in this area in different trial populations. Additionally, those who use social media in recruitment report a significant impact of the format and content of advertisements, some reporting success with videos over images, others reporting Instagram over Facebook [20-22]. Given the complexities of social media marketing, there are queries about whether it is worth the need to train researchers in social media processes or recruit social media professionals [23].

It is clear that, given this is a relatively new and ever-changing methodology, more research needs to be done to maximize its effectiveness. Reuter [24] argues that for clinical trials to harness social media recruitment, we need more data on whether social media is better suited for the recruitment of study participants from certain demographic groups and the characteristics of effective messaging approaches. Additionally, she argues that we need to explore the effect of social media compared with other approaches on consent. Hence, we have two aims for this paper: (1) to provide a pragmatic depiction of co-designing and implementing a social media strategy with our advisory group, including the link between social media content and consent rate, and (2) to compare the demographic information of participants who were recruited via social media and other methods. We hope that our findings will support learning for future trials and researchers to implement social media recruitment with maximum efficiency.

## Methods

### Overview

In this paper, we conducted an observational analysis of social media as a recruitment method in a nested study within 2 mental health research trials aimed at university students. These trials are part of the Nurture-U research project [25] and test 2 separate interventions (see trial protocols for details):

1. “Reducing Worry”: an app to reduce worry and boost confidence. This trial is preventative, aimed at people with worry, but no clinical symptoms of anxiety or depression. Participants are randomized to test the app or continue receiving treatment as usual (ISRCTN86795807) [26].
2. “Online CBT”: a comparison of therapist-guided versus self-guided online cognitive-behavioral therapy, aimed at students with clinical levels of anxiety or depression. Participants are randomized to one of the 2 types of therapy (ISRCTN56784470).

The links from the social media adverts led to the Nurture-U trials webpage, which linked to the REDCap (Research Electronic Data Capture; Vanderbilt University) trials software [27]. The recruitment process was designed so that all potential participants were directed to one screening questionnaire on REDCap. Participants were screened using the Patient Health Questionnaire-9 [28], Generalized Anxiety Disorder-7 questionnaires [29], the Penn State Worry Questionnaire-short form [30], and the Ruminative Response Scale-Brooding [31]. If their scores indicated clinical anxiety or depression, they were directed to the information sheets and consent form for the online CBT trial. If not, but they indicated high levels of worry and rumination, they were directed to the Reducing Worry trial. There was one more trial in Nurture-U (ISRCTN18276230), but this recruited only from the University of Exeter and did not use social media for recruitment. Reducing Worry and Online CBT were recruited nationally, and hence, for consistency, we report only on the Reducing Worry and online CBT trials for this paper.

The Nurture-U project began in December 2021, with an 18-month intervention-development stage before trials began in July 2023. This paper includes an analysis of the social media recruitment from when we started using paid adverts in November 2023 to the end of the Reducing Worry recruitment in December 2024.

### Developing a Social Media Strategy With the Nurture-U Student Advisory Group

The Nurture-U student advisory group (SAG) has been engaged in every stage of the project from its inception. More details on the role of the SAG in the first year of the project have been published elsewhere [32].

The SAG developed Nurture-U’s social media strategy. This involved initial meetings in Summer 2022, where Instagram was decided as the platform to focus on, and a plan to provide educational content alongside promotional material was made to encourage engagement. While accounts were created on X and Facebook, these accounts were not active beyond occasional promotional posts.

Instagram followers increased organically through promotion at Freshers Fairs in the participating universities. Engagement increased more actively through the summer and autumn of 2023 as student advisors led on creating and posting more student-generated content. At the time of writing, the project has 1087 followers on Instagram. Since Autumn 2023, on advice from the SAG, Nurture-U also launched TikTok and LinkedIn accounts. Video content generated by students and the research team is posted on both Instagram and TikTok, and LinkedIn is focused on making connections with both students and academics in the field.

The strategy for trial recruitment was developed with the SAG as a rolling point of discussion across termly SAG meetings in 2023 and 2024. The ad co-designing process with student advisors used tools like Mural and Canva to gain student advisory feedback; student advisors could both vote on our initial concepts (that included critical wording specific to the research) as well as rework the designs or create their own. We also explored market research options, taking note of what other research trials were promoting. The research team then developed and tested different adverts.

### Data Collection and Analysis

We kept a record of the content of each advert we trialed, price per day, and duration on a Microsoft Excel spreadsheet. We collected data from Meta and TikTok business platforms, Google Analytics, and the REDCap platform we were using for Trials recruitment.

The recruitment journey and corresponding data collection points were as follows:

1. Potential participant clicking on the advert (Meta and TikTok “link clicks” data from business pages).
2. Potential participant landing on our “Take Part Now” Trials page of the Nurture-U website (Google Analytics).
3. Potential participant consenting to screening questionnaire to ascertain eligibility for trial participation (REDCap software).
4. Enrolled participant consenting to the Reducing Worry or online CBT trial (REDCap software).

The REDCap screening questionnaire included the question “Where did you find out about the Nurture-U project and this intervention study?.” Students could tick “Instagram,” “Facebook,” “TikTok,” “Twitter,” or “other social media,” as well as the other recruitment methods such as “University email,” “Lecture shout out,” or “Poster.” We extracted the numbers who came into the screening database through the social media platforms we were advertising on each week to ascertain the success of the adverts. The REDCap consenting data also recorded participant location based on their university email, which enabled us to ascertain whether particular location-based advertising was effective.

All the data was input into an Excel spreadsheet. A descriptive analysis of the performance of the advertising over time was conducted using line graphs.

The REDCap data for all participants who consented to take part in the study were imported into R for analysis. Of the participants who consented to the trials, 46% (2081/4387) did not complete the baseline demographic questionnaires required for randomization and were therefore excluded from this analysis. As the aim of the study was to compare the demographics of participants who ultimately took part in the trials, and given that the missing data were due to dropout, we conducted a complete case analysis of those who had answered the demographic questionnaires. Multiple imputation was not considered appropriate, as the missingness was not at random and related to nonparticipation.

The answers to the REDCap question “Where did you find out about the Nurture-U project and this intervention study?” allowed us to create 2 groups of participants: those who had been recruited through social media and those who had not. Independent sample *t* tests were used to see if there were significant differences between the participants based on age. Chi-square was used to explore whether there was a significant difference between the sample recruited through social media and those recruited through other methods, according to ethnicity, gender, sexual orientation, disability, and university attended. Participants reported their ethnicity as one of 18 options; these were combined into “Asian,” “Black,” “Mixed or Other,” and “White” to allow comparison between larger groups. Those who reported “Prefer Not to Say” or “Not Known” were excluded from the analysis (n=8). Participants reported on their gender as “Female,” “Male,” and “Gender diverse.” “Prefer Not to Say” and “Prefer to Self Describe” were excluded from the analysis due to small numbers (n=17). Also, due to small numbers in some of the categories, the 7 possible responses for sexual orientation were collapsed into “LGBTQIA+” and “Heterosexual.” Students reported their disability status as binary “yes” or “no response.” The university students attended were elicited from their email, and these were combined into 2 groups according to whether they were from a Nurture-U partner university (Cardiff University; King’s College London; Newcastle University; and the Universities of Exeter, Oxford, and Southampton) or another university. The significance level for all analyses was set at *α*=.05. This study was reported in accordance with the STROBE (Strengthening the Reporting of Observational Studies in Epidemiology) guidelines for cohort, case-control, and cross-sectional studies (combined).

### Ethical Considerations

Ethics approval for all Nurture-U trials was obtained from the University of Exeter Psychology Research Ethics Committee (institutional review board numbers: 523095; 523085). All participants in the REDCap dataset gave informed consent, and no personal data were visible to the researchers. Data collected from social media platforms contains no personally identifiable information; the data is only in the form of the number of clicks and views. Participants were remunerated for completing follow-up questionnaires as part of the Nurture-U trials (up to a total of £30 [US $40.53]), but did not receive payment at trial entry, which is the data used in this study.

## Results

### Development of Social Media Strategy

Ten different adverts were trialed over the 12-month period; a screenshot of the first page of each advert can be found in Table 1.

**Table 1.** Social media advert content, duration, costs, and corresponding recruitment figures for university student mental health trials.

	Description	First page design	Months live	Cost per day (US $)	Numbers consented to screening	Numbers recruited to trial participation
A	Canva reel with music: Design from student cocreated poster mirroring successful campaign in previous trial.	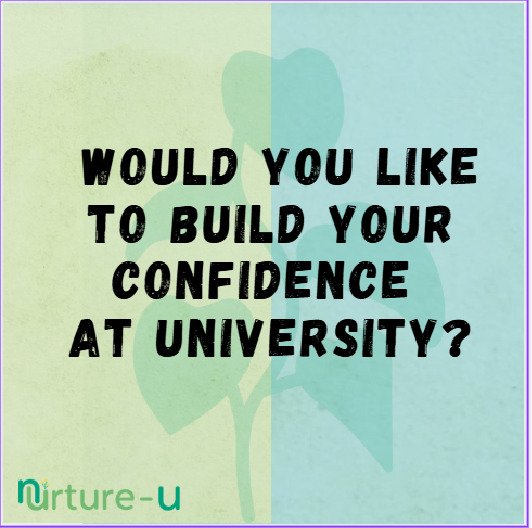	Nov-Dec 2023	6	0	0
B	Location-specific student callout reel (trialed in Glasgow, Leeds, Bristol, Dundee, Nottingham, and Sheffield).	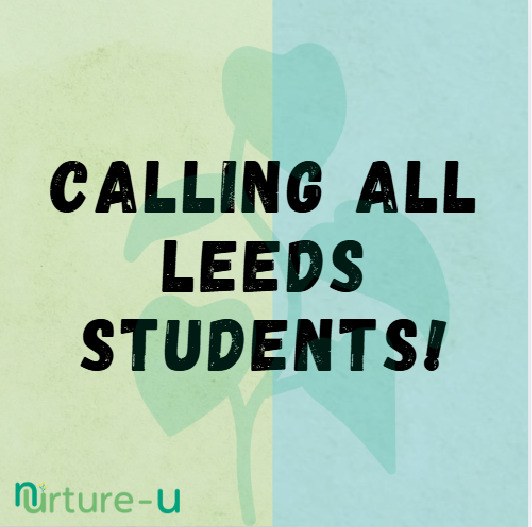	Dec 23 Feb 24 (alongside D)	12.50 6	22 See D below	8 See D below
C	Cocreated advert with student advisors—students talking to camera.	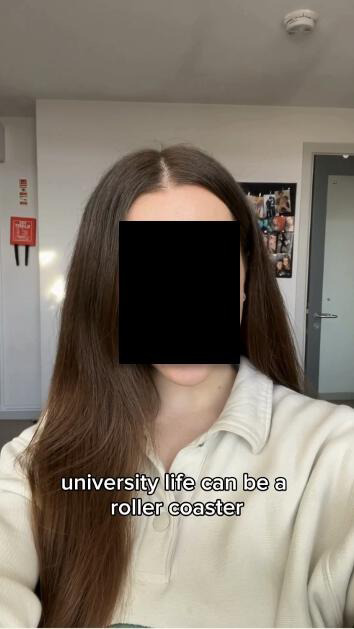	Feb 24 Mar 24	6 10	0 6	0 2
D	Student talking to camera without script.	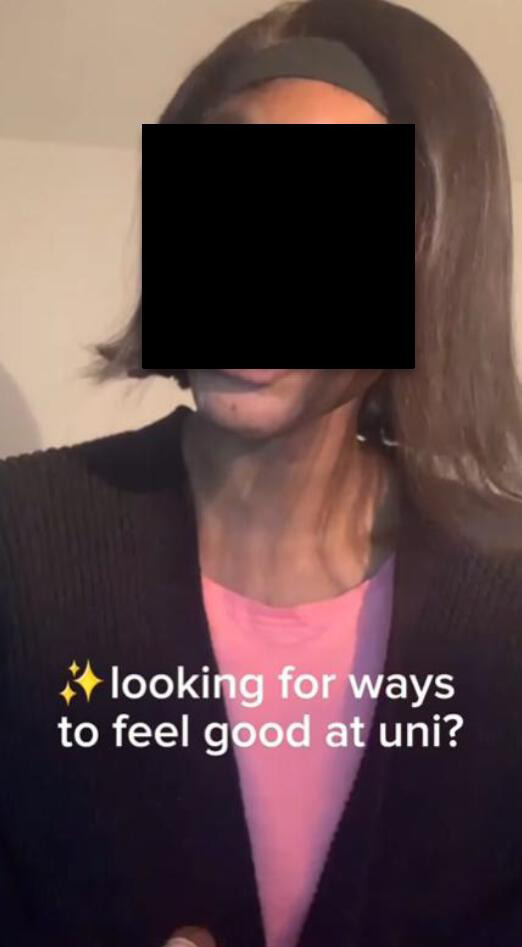	Feb 24 (alongside B)	6	8	4
E	“Build your confidence” carousel post—different university logos according to location. Location details are provided in Table 2.	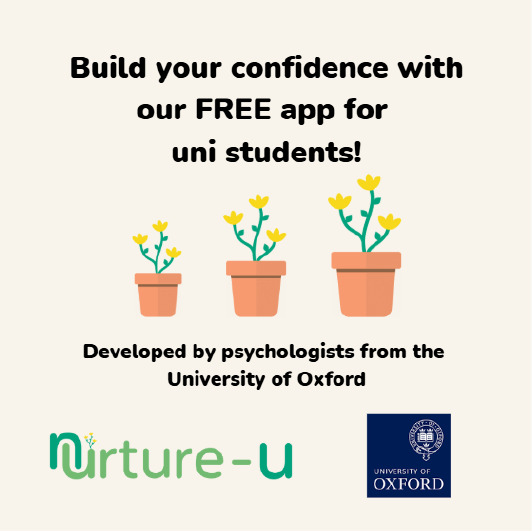	Mar – Jun 24 Jul – Aug 24 (alongside F) Aug 24 (alongside G) Sep 24 Oct 24 (alongside H) Nov 24 (alongside I) Dec 24 (alongside J)	70 70 70 140 70 70 40-80	578 See F below See G below 70 See H below See I below See J below	282 See F below See G below 31 See H below See I below See J below
F	Summer carousel post.	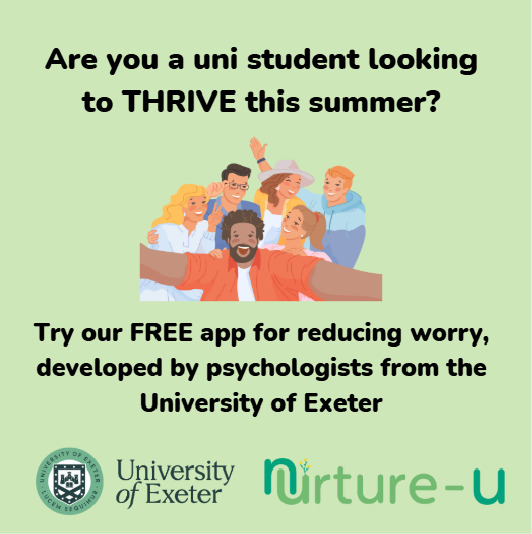	Jul – Aug 24 (alongside E)	70	31	8
G	Worry carousel post.	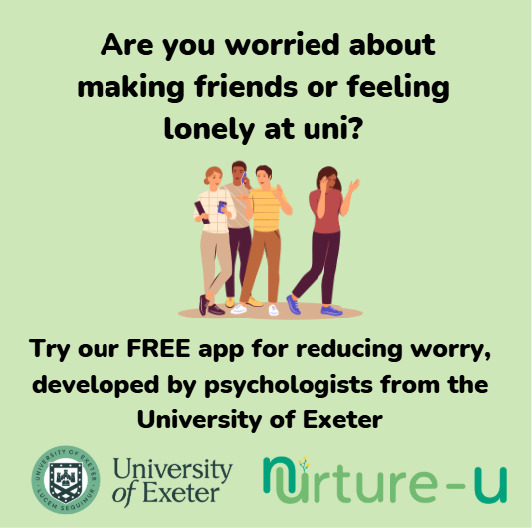	Aug 24 (alongside E)	70	2	1
H	Rainbow carousel post.	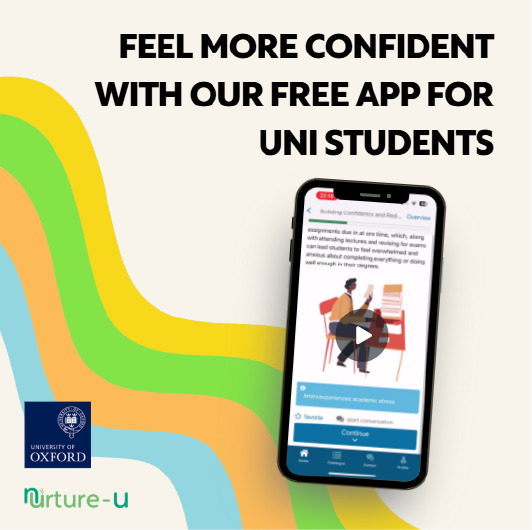	Oct 24 (alongside E and Instagram Story ad with same design)	70	57	24
I	Overthinking carousel post.	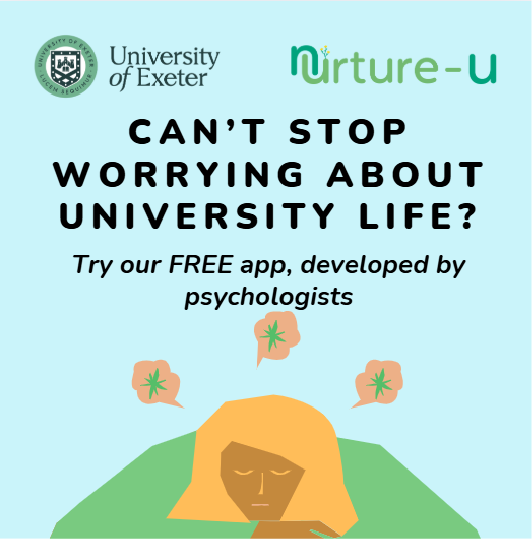	Nov 24 (alongside E)	70	19	4
J	3 weeks left carousel post.	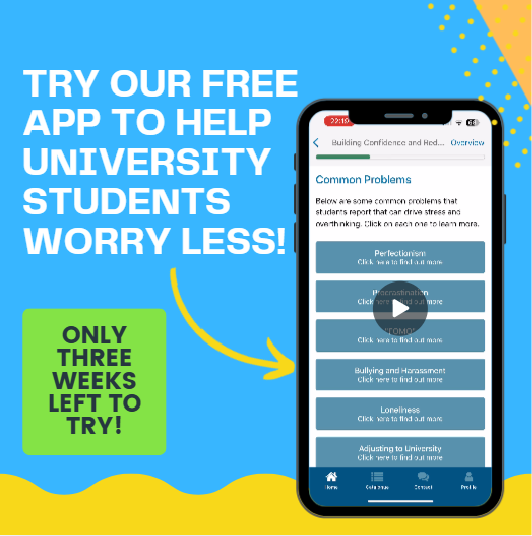	Dec 24 (alongside E)	70	15	6

**Table 2.** Total link clicks on social media adverts for recruitment to the university student mental health trial by advert location.

Location	Number of link clicks	Numbers consented to participation
London, Cambridge, Colchester (South East)	9718	186
Oxford, Birmingham, Reading, Staffordshire (South/Midlands)	8578	102
Leeds, Manchester, Newcastle, Nottingham, Sheffield, York (North)	6878	277
Scotland	5881	107
Wales	5728	134
Southampton, Bournemouth, Brighton (South)	5387	93
Exeter, Bristol, Plymouth, Falmouth (South West)	5342	172

### Advertising Platform

The Meta advert did not specify which Meta platform, but 95% (n=748) of the 842 students who consented to participation from social media reported seeing an advert on Instagram. It was not possible to tell from the Meta business suite where our advert was primarily placed, so it is not clear whether Instagram was more successful than Facebook or whether Meta chose to place more adverts on Instagram.

**Figure 1.**
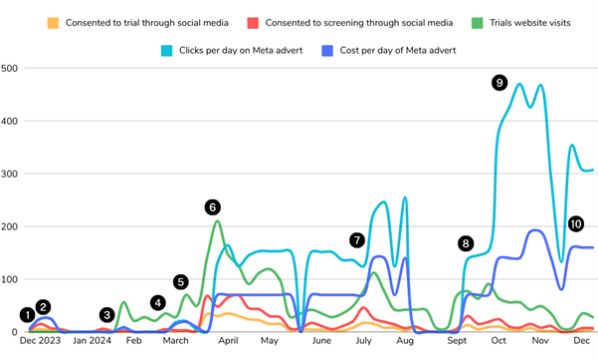
Shows the impact over time of advert content, broken down by link clicks, numbers consenting to screening, and numbers consenting to trial participation. As demonstrated by the spike in activity at point 6 on the graph, Advert E was initially extremely successful: an average of 63 students per week consented to screening, and 31 per week consented to trial participation and began the randomization process over the first 4 weeks. However, the number of participants consenting started steadily decreasing as the summer approached, despite the cost of the adverts and Meta’s reports of clicks remaining consistent. We surmised that this might be due to students having exams and then finishing their university studies for the summer. Despite SAG-designed changes to content to reflect this (Table 1 **F,G**), recruitment declined, and we paused adverts in August. As the new academic year began, recruitment rose again with a decline towards the end of term, despite new content and increased spend (Table 1 **H-J**).

The SAG advised us not to advertise on TikTok, citing minimal engagement with adverts on that platform. We trialed 2 reels (Figure 1 C,D) on TikTok across February. However, this resulted in 2 students consenting to screening, and neither of these consented to take part in the studies. Hence, we did not continue.

We did not pay for advertising on X, as undergraduate SAG members indicated that they did not use this platform. However, we did promote Nurture-U more generally across the year on X, and one participant indicated they had entered REDCap through a link on an X post.

### Advertising Content

Table 1 shows the content, dates run, costs, and numbers recruited in those dates. Figure 1 allows for more in-depth comparison over time, displaying (1) average of link clicks per day, (2) costs of each advert per day, (3) average numbers who landed on the Trials page of our website per day, (4) the numbers who consented to screening of the trials and (5) numbers randomized over the course of the recruitment period. The numbers in black circles indicate what changes were made at each stage in recruitment in this timeline, corresponding to Figure 1.

An initial reel (Table 1A) mirrored advertisements from a previous trial in 2019 [26]: a slideshow with text that played as a video with music. These initially were not successful, but location-specific designs (B) with higher budgets had more success. Advertisement was paused over the Christmas period and January to focus on local recruitment and plan a social media strategy with our SAG. This resulted in 2 reels using selfie mode with students talking to the camera, cocreated with student advisors (C+D). These were not successful in boosting recruitment.

A new design (E) was developed based on the following SAG input:

- Simple designs are important: People will just keep scrolling if they have to work out what they are looking at.
- Credibility is key: We need to be clear that we are from universities and not a potential sham company.
- Transparency: A clear example of the “product” or software in use will encourage people to click.
- Colorful or aesthetically pleasing images to support the text will also stop people from scrolling.

Carousel posts were implemented instead of reels, based on market research of other mental health apps and well-being advertising. We decided to focus the content of the adverts on the Reducing Worry app, hypothesizing that this was harder to recruit for than therapy. These core design decisions remained central to subsequent designs (Table 1 F-J), with slight changes in content and color according to time of year.

### Implementation and Success of Social Media Strategy

#### Engagement and Recruitment Over Time

While there was attrition between consenting for eligibility screening and consenting to participation throughout the year, Figure 1 shows the same pattern in which numbers rise and fall, indicating that both metrics are responding proportionally to the external influences. These are most likely the time of year and advert content, given the money spent on the adverts remained the same or increased. In July and October, Meta reports of link clicks continued rising with the increase in cost-per-day of the advert, but the consent to screening and participation continued falling (points 7 and 9 on Figure 1). Hence, there was some discrepancy between Meta reports of successful advert engagement and resulting recruitment success.

#### Engagement and Recruitment by Location

Table 2 shows the total link clicks and numbers consented to participation of each location over the whole recruitment period. London was the most successful area for link clicks but had comparable recruitment success to the South West, which had 55% of the total link clicks of London and the South East. There were higher numbers of link clicks in Oxford and the Midlands compared with the larger university cities in the North of England, but the Northern location had the most recruited participants. The more sparsely populated south-central and west areas were the least successful on link clicks and recruitment numbers.

### Comparison of the Sample

Table 3 details the comparisons between the sample collected through social media and the sample collected through other methods. There were 2369 students who consented to be trial participants in the observed timeframe. A total of 842 participants (35.6%) indicated they had been recruited through a social media advert, 1527 (64.4%) participants had been recruited through other methods. There was a 46% drop out after consent, meaning a total of 1329 participants completed demographic questionnaires and were randomized to trials. Of these, 554 (42%) were recruited through social media, and 775 (58%) were recruited through other methods.

**Table 3.** Comparison of the sample recruited through social media and other methods.

Variables	Social media (n=554)	Other methods (n=775)	Comparison
Age (years)			
Mean (SD)	21.25 (2.63)	26.45 (7.93)	—^a^
Mean difference (SE)	—	—	−3.49 (0.31)^b^
*t* test (*df*)	—	—	15.146 (1927.5)
95% CI	21.06392 to 21.43937	24.33245 to 25.15566	−3.040178 to −3.944640
Ethnicity			
White, n (%)	280 (80.4)	504 (75.9)	—
Other, n (%)	68 (19.6)	160 (24.1)	—
Chi-square (*df*)	—	—	2.4609 (1)
Gender			
Female, n (%)	308 (83.3)	582 (81.9)	—
Male, n (%)	35 (9.4)	99 (13.9)	—
Gender diverse, n (%)	27 (7.3)	30 (4.2)	—
Chi-square (*df*)	—	—	8.3422 (2)^c^
Sexual orientation			
Heterosexual, n (%)	171 (48.7)	420 (62.7)	—
LGBTQIA+, n (%)	180 (51.3)	250 (37.3)	—
Chi-square (*df*)	—	—	17.867 (1)^b^
Disability			
Disabled, n (%)	103 (27.5)	154 (21.3)	—
Nondisabled, n (%)	272 (72.5)	569 (78.7)	—
Chi-square (*df*)	—	—	4.8992 (1)^c^
University			
Partner university, n (%)	235 (31.0)	999 (69.8)	—
Nonpartner university, n (%)	524 (69.0)	433 (30.2)	—
Chi-square (*df*)	—	—	432.33 (1)^b^

a Not applicable.

b *P*<.001.

c *P*<.05.

Those recruited through social media were significantly younger than those recruited through other methods (*t*_1927.5_=15.15, *P*<.001). Participants recruited through social media had a mean age of 21.25 (SD 2.63, 95% CI 21.06‐21.44) years compared with 26.45 (SD 7.93, 95% CI 24.33‐25.156) years. There was a mean difference of −3.49 years (SE=0.31, 95% CI –3.041 to –3.945).

Due to small numbers of Black (n=31) and Mixed or Other students (n=77), these categories were combined into “White” and “Other Ethnic Group.” Of those who answered the ethnicity questions, the majority were White (728/1011, 72%). There was no significant difference between the social media and other methods group according to this variable (*χ*²_1_=2.4609, *P*=.12).

There was a significant difference in the samples according to gender (*χ*²_2_=8.34, *P*=.02). In the sample recruited through social media, 83.3% (n=308) were female, 9.4% (n=35) were male, and 7.3% (n=27) were gender diverse. The split in the sample recruited through other methods was 81.9% (n=582), 13.9% (n=99), and 4.2% (n=30), respectively. Hence, the differences appear to lie in there being more gender-diverse students recruited through social media compared with other methods (27/370, 7%, vs 30/711, 4%, CI 3.4%‐13.7%), but fewer male students (35/370, 4%, 95% CI [3.4%‐13.7%] vs 99/711, 7%, 95% CI [1.6%‐9.8%]). A significantly higher proportion of students in the social media sample were from the LGBTQIA+ community (180/351, 51%, 95% CI [41.3%‐60.6%] vs 350/711, 37%, 95% CI [28.2%‐46.8%]; *χ*²_1_=17.87, *P*<.001). There was also a significant difference in the sample reporting a disability (103/375, 27.5%, 95% CI [19.7%‐37.0%] vs 154/723, 21.3%, 95% CI [14.4%‐30.3%]; *χ*²_1_=4.90, *P*=.03).

Of participants recruited through social media, 69% (n=524) were from universities that were not the 6 Nurture-U partner universities, compared with 30.2% (n=433) of those from other methods (*χ*²_1_=302.01, *P*<.001).

## Discussion

### Principal Findings

Our observational study of the effect of trial recruitment methods on participant characteristics had important implications for planning future trials, highlighting the necessity to build in multiple recruitment approaches in order to ensure a diverse sample. The social media sample was younger and had a higher proportion of LGBTQIA+, disabled, and gender-diverse students, while male participants represented a larger proportion of the sample in those recruited through other methods. Co-designing a social media recruitment strategy allowed us to create advertising content that was appealing to students but still necessitated trial and error in establishing what formats enabled successful engagement and recruitment on different platforms. Alongside content, time of year was the key factor in successful campaigns, with recruitment numbers fluctuating alongside student engagement with the university in the academic year.

Our pragmatic description of how our recruitment fluctuated according to time of year and advert content shows the importance of co-designing recruitment strategies for each project, not relying on the research team’s experience on previous projects. The simple, bright colors and large lettering of the successful adverts for the previous mental health trial, EcoWeb [26], were not successful for Nurture-U. There were 4 years between recruitment periods for the 2 trials, and in this time, there had been a marked shift in social media trends. The rise of TikTok and the increase in online interactions over the COVID-19 pandemic have led to societal awareness of the differences in social media platforms and the roles each one plays in our online lives [33]. Our student advisors were clear that Instagram would be the most effective platform for our advertising, and they were right. The fact that a simple carousel post was the most successful over more interactive videos and reels may reflect what people want from Instagram, and hence what people want from an Instagram advert. A research study’s Instagram profile has been shown to affect participation [34], showing how important the social media presence of a study or research group can be on people’s attitudes towards participation. Our SAG led our successful Instagram output, through which we have organically gained over a thousand followers through “day in the life” reels and student-focused wellbeing advice, and have built up a respectable level of TikTok engagement with similar content. However, similar approaches to our advertising content, ie, students speaking to the camera, were not successful. This demonstrates the difference between what works as content and what works as an advert and emphasizes the importance of effective market research.

Previous literature experimenting with different content for trial recruitment adverts has shown that using doctors to talk about clinical trials boosts recruitment as compared with using trial participants [14] and that information shared by a doctor may be more effective in disseminating formal and medical information and tackling logistical barriers to health [35]. We did not attempt to use an expert clinical voice as a method, and this may have been an omission. Our student advisors suggested applying to student influencers as prominent voices in the online space. We approached several suggested accounts, but we were not successful, and those who did respond charged very high fees. We believe there are 2 reasons for this. One is that people’s profiles are carefully curated so that they are very specific in what they promote [36]. Second, and related, there remain negative conversations around these, which individuals may want to avoid. A recent study of reactions to influencers’ posts about mental health found 25% of the comments were negative in content and 20% contained mental health stigma [37].

On reflection, a limitation of our co-design process with students is that we focused on content rather than on how we compete for student attention with external factors such as exams and university holidays. Our lack of planning for this meant we were left with only small windows for higher recruitment, specifically the beginning of academic terms. There is little research that has examined the influence of seasonality on recruitment. Langlois and Kriglstein [38] attributed not reaching participant targets in an online survey of Esport players partly to the recruitment period falling over the summer months, and there’s acknowledgment of a reduction in clinical trial recruitment in the summer due to staff holidays in hospital settings [39]. While the student calendar is particularly variable and marked by different stages of activity, the wider population will also be affected by the time of year and corresponding variations in engagement in social media [40]. This is particularly challenging for research trials where recruitment windows are short, and further research is needed into the best methods to combat these fluctuations.

There were resource issues throughout the recruitment period that impacted our success, which have also been described by other research teams [23,40,41]. It was incredibly time-intensive for a research team inexperienced in creating paid advertising campaigns to both design and implement our recruitment strategy. There are a wide variety of ways to advertise on Meta alone. Our lack of expertise is also related to experimenting with different platforms. Our attempt at advertising on TikTok partly failed due to needing a TikTok Business account to enable links in videos and the challenges of navigating this with limited time and financial resources. As mentioned above, social media adverts will be more successful if they are linked to active and engaged accounts that post content regularly, which requires additional resources to maintain. There is no doubt that our costs were higher than they could have been if the team had known how to be more strategic in social media recruitment. There are published guides for social media recruitment to research, although many prioritize privacy and ethical concerns over marketing strategies [42]. Hence, studies that need to maximize the effectiveness of social media as a key recruitment strategy (for example, due to short recruitment windows or lack of other options for recruitment) may wish to use the services of a specialist company [42].

Social media strategy can only impact recruitment to a limited extent: once people have clicked the links, it is the study website and online data collection methods that are key for retention. Complexity and length of the screening process for clinical trials have previously been identified as a key barrier to recruitment [43]. The Nurture-U trials were set up to target widely and then siphon people into the appropriate trial based on the level of symptoms in the screening questionnaire. However, it takes a minimum of 10 minutes from the start of the screening process to consenting and being randomized for a trial. While this may not be considered a lengthy screening process for a clinical trial where participants are recruited in a hospital or university, marketing analytics indicate that the conversion rate from social media advert to website engagement can be as low as 1%‐9% [44]. Hence, 10 minutes is a big ask for people who have clicked through from a social media advert, considering they may be scrolling through Instagram while traveling, before a lecture, or in social situations. This is reflected in the reduction between the numbers in our REDCap database and those who consented to screening and then randomization.

Additionally, there was a challenge of advertising 2 trials in one. One trial contained an opportunity for therapy, and the other an app, and it was difficult to portray both options in a simple way in a social media advert. This led us to focus our content on the Reducing Worry app, our rationale being that it was under-recruiting. However, the unknown nature of the content of this app and its efficacy is likely to have affected the success of our advertising strategy [45]. Most trials of health-based apps recruit largely through clinical populations, targeting specific groups of people with specific symptoms or health goals, often through hospital settings [46]. We were aiming to recruit from a general university population and to prevent rather than treat symptoms. Trials of preventative interventions, recruiting people at risk of conditions but who do not have them yet, are far harder to recruit to than those testing different treatment options of an established condition [47].

There are many papers that cite the value of social media for widening the reach of adverts for research, but little research that directly compares the sample that was recruited through social media to those who were recruited through other methods [4,8]. Social media can increase representativeness through targeting campaigns to specific demographics, but by its nature, it will only reach those who use chosen platforms [23]. There are concerns that the more research recruitment takes place on social media, the more certain groups will be excluded, such as older participants or participants from specific ethnic groups [6]. Despite this, many studies report the benefits of recruiting hard-to-reach populations through social media [42]. This was also a key success of our strategy, in that there was a higher proportion of students from universities that were not part of Nurture-U in the sample recruited from social media. These students were harder to reach through other methods due to limited abilities to share study details in universities where the team was not based. Additionally, as the Nurture-U universities are all Russell Group universities, a group of universities with high research focuses requiring high grades to attend, increasing the breadth of universities involved in the project was a key priority to diversify our trial participants. When looking at link clicks by location, there were more link clicks in higher population areas such as London, but on examining the numbers consistently by location, it was evident that the more successful locations were those with large university student populations, for example, in the North of England. This is an important factor to keep in mind when planning social media strategies, that it is better to target locations according to your specific population, rather than larger general population numbers.

Many studies reported comparatively more females and younger participants recruited through social media [48]. We also had younger participants, likely explained by the 18‐25 year age restriction on our adverts. Women are 3 times more likely to have a mental health diagnosis than men in the 16‐24 year age group [49], which may explain our high female sample. While women are more likely to spend more time on social media, which leads to proportionally more impact on mental health [50], the use of social media compared with other methods did not appear to impact the proportion of females in our project. Interestingly, there was a larger contingent of males in the other recruitment methods, and a higher proportion of gender-diverse or self-defining students recruited from social media. This highlights the importance of using a mix of methods to enable a diverse sample.

There was no significant impact of social media on the ethnic diversity of our sample, which differs from other studies showing differences in ethnicity according to method [51]. There were, however, more students from the LGBTQIA+ and disabled communities recruited through social media compared with other methods. This finding builds on previous research that demonstrates social media as making it easier to recruit from this population, especially those who are less able to connect face-to-face, for example, due to living in a rural location or having impaired mobility [52,53]. The digital world can be a safe and supportive space for LGBTQIA+ and disabled youth, allowing exploration of identity [53,54]. It may be both that LGBTQIA+ and disabled students are more active on social media, but also that they interact with accounts that are similar to Nurture-U, with emphasis on well-being and community, meaning the algorithm pushed out advert more towards this sample. It is worth noting that social media can be a “double edged sword” for this community, with many experiencing discrimination and rejection [55], and these risks can extend to those researching in this space [56].

There are aspects of recruiting through social media that we did not explore in this study. There are ethical issues in social media advertising, including the lack of control over how posts are shared, what people comment, and privacy and data protection for those who interact with the adverts [57]. For example, we chose to focus on the Reducing Worry trial in advertising, which may have impacted people’s understanding if they were directed to the Online CBT study. In future research, it would be useful to include a measure to capture these issues, such as recording engagement or people’s understanding of the study messaging. Additionally, our study used post hoc comparisons; it was not designed specifically for a rigorous analysis and comparison of the success of different recruitment methods. A more robust study design, for example, an ABA design with direct comparison of different adverts, would provide stronger evidence of the efficacy of different approaches. Additionally, the ability to control for the impact of time of year, especially in the university population, would allow a more definitive exploration of the impact of advert content. Last, we are aware that, in recruiting university students, we are directly targeting a younger population who are more likely to be both engaged in social media and, as a result of growing up with it, more critical in their use. Both these factors will have implications for interpretation and generalizability for other settings and populations.

### Conclusions

Our study is innovative in that it is one of the first studies that provides a description of how different recruitment approaches allow for differences in participant characteristics in clinical trials, alongside a pragmatic depiction of the challenges in implementing a co-designed recruitment strategy in a university setting. Our study demonstrates the positive effect of social media in accounting for a large proportion of our recruitment numbers and providing a higher proportion of gender-diverse and LGBTQIA+ students than other methods. However, the fact that other methods enabled more success in recruiting males, who are underrepresented in mental health research, demonstrates the importance of using diverse recruitment strategies. Our study also provided a pragmatic description of the co-design of a social media strategy, contributing to the literature by providing evidence of the importance of building time for adaptation of content and timing of social media adverts depending on your population within your research process. However, there are many challenges to the successful implementation of a social media recruitment campaign, including content design, recruitment timelines, resources available, and the ever-evolving internet landscape. Coproduction with those whom the research project is targeting, in our case, students, is key to a successful recruitment strategy. Our study contributes to the field by providing research evidence on the efficacy of different recruitment strategies for planning future trials. Our key real-world recommendation is to allow time and resources for planning multiple recruitment strategies in order to ensure a diverse range of participants take part in research.

## Abbreviations

REDCap: Research Electronic Data Capture
SAG: student advisory group
STROBE: Strengthening the Reporting of Observational Studies in Epidemiology
